# A Hospital-Based Longitudinal Study of Rubber Band Ligation and Sclerotherapy Treatment for Internal Hemorrhoids From South India

**DOI:** 10.7759/cureus.64570

**Published:** 2024-07-15

**Authors:** Samrobinson J, Jeyaganesh R, Geetha Arumugam, Nawin J Vignesh

**Affiliations:** 1 General Surgery, Vels Medical College and Hospital, Tiruvallur, IND; 2 General Surgery, Velammal Medical College and Hospital, Madurai, IND; 3 Community Medicine, Karpagam Faculty of Medical Sciences and Research, Coimbatore, IND

**Keywords:** piles, endovenous chemical ablation, sclerotherapy, banding, hemorrhoids

## Abstract

Background

Hemorrhoids are an extremely common surgical condition affecting millions of individuals worldwide. Treatment options for hemorrhoids vary depending on the severity of symptoms and the type of hemorrhoids. The common non-surgical procedures for grade one and two hemorrhoids include rubber band ligation and sclerotherapy. The present study aims to compare the efficiency of rubber band ligation and sclerotherapy for the treatment of symptomatic grade one and two internal hemorrhoids in a tertiary care hospital.

Methodology

We conducted a one-year longitudinal survey among 200 patients with internal hemorrhoids in a tertiary care center in Madurai. We gathered data on demographic profiles, symptoms, postoperative complications, intraoperative pain, and treatment outcomes. Data analysis was done using the Pearson chi-square test to assess the difference between rubber band ligation and sclerotherapy treatment groups. A p-value <0.05 was considered statistically significant.

Results

A total of 200 patients were studied, of whom 100 belonged to the rubber band ligation treatment group and 100 belonged to the sclerotherapy treatment group. The preoperative symptoms were similarly distributed between both treatment groups. Intraoperative and immediate postoperative pain was higher in the rubber band ligation group than in the sclerotherapy group. Post-procedure complications were more commonly seen in the rubber band ligation group than in the sclerotherapy group at various weeks of the procedures.

Conclusions

Postoperative complications such as bleeding, prolapse, and infection/discharge were significantly different between the two treatment groups. The treatment outcome was significantly different between the two treatment groups after three, six, and nine weeks postoperatively. Overall, the sclerotherapy group was associated with fewer postoperative complications, more excellent patient response, and a more complete response to treatment than the rubber band ligation group.

## Introduction

Hemorrhoids, though commonly misunderstood or overlooked, are an extremely common surgical condition affecting millions of individuals worldwide. These swollen and inflamed veins in the rectum and anus can cause discomfort, pain, and even bleeding, significantly impacting one’s quality of life. Despite their prevalence, discussing hemorrhoids can often be met with discomfort or embarrassment, leading many to suffer in silence without seeking proper medical attention.

The estimates of hemorrhoids by various studies suggest that they affect a significant proportion of the Indian population. According to Safir et al., approximately one in every four people over the age of 30 suffers from hemorrhoids [1]. Several studies have observed similar prevalence rates in Western and Indian studies [2-4].

Treatment options for hemorrhoids vary depending on the severity of symptoms and the type of hemorrhoids. Some treatment options include home remedies, medication, non-surgical procedures, and surgical procedures. Among them, non-surgical procedures are often considered the first line of treatment as they are minimally invasive, have a low risk of complications, and are an effective outpatient procedure for mild-to-moderate cases.

The common non-surgical procedures for grade one and two hemorrhoids include rubber band ligation and sclerotherapy. Based on a meta-analysis, the rubber band ligation appeared to have the lowest incidence of recurrent symptoms and the need for retreatment [5]. Rubber band ligation is also the most popular non-surgical intervention for hemorrhoids performed by surgeons [6]. It is a relatively safe and painless procedure with minimal complications. However, it is contraindicated in patients on anticoagulants or with bleeding disorders, as well as those with concurrent anorectal sepsis. In such situations, injection sclerotherapy is an effective and safe procedure for treating bleeding hemorrhoids. However, results from a systematic review found no significant difference in the effectiveness of rubber band ligation and sclerotherapy in treating internal hemorrhoids in terms of blood loss, recurrences, and complications [7,8]. Studies related to the above-mentioned treatment are scarce in Indian settings and therefore the reliability of the data is affected. Therefore, the present study aims to compare the efficiency of rubber band ligation and sclerotherapy for the treatment of symptomatic grade one and two internal hemorrhoids in a tertiary care hospital.

## Materials and methods

Study design and period

We conducted a comparative longitudinal study in the surgical procedure room for outpatients in the general surgery department of a tertiary care hospital situated in Madurai, Tamil Nadu, India. The study was conducted for one year from April 2021 to May 2022.

Study site

According to the previous year’s census, an average of 30 hemorrhoid cases are reported in a month in the general surgery department of the tertiary care hospital. While the reported hemorrhoid cases comprise all grades, the present study included cases of only grade one and two internal hemorrhoids.

Sample size calculation

The sample size was calculated using a sample proportion sample size formula. According to a study conducted by Nasir et al. in Pakistan, the complete recovery of second-degree hemorrhoids by sclerotherapy was 55.1% and the complete recovery of second-degree hemorrhoids by rubber band ligation was 75.95% [9]. Considering a 95% confidence interval with a margin of error of 5% and with a power of 80%, the sample size obtained was 82 for each group. Therefore, the total calculated sample size was 164.

Sampling method

Patients visiting the outpatient ward of the general surgery department in a tertiary care hospital were recruited. A detailed history was obtained from all recruited patients with emphasis on symptoms, occupation, and dietary habits of the patients. All included patients underwent digital rectal examination and proctoscopy. Convenient sampling was used where all patients with grade one and two hemorrhoids were included until the sum of 100 was obtained in each group during the data collection period. The choice of treatment was based on patients’ preference whereby they were assigned to either of the two treatment groups, i.e., sclerotherapy or rubber band ligation.

Inclusion and exclusion criteria

The study participants were patients with first and second-degree hemorrhoids who presented with bleeding per rectum with or without associated symptoms such as mucosal prolapse, discharge, pruritus, and pain, as well as having been diagnosed on history and proctoscopy findings such as visible bleeding and engorged anal cushions. Both male and female patients who were above the age of 20 years were included in the study. Any patient with bleeding diathesis or on anticoagulants were excluded. Patients having anal fissures and/or perianal abscesses, pregnant women, or those having any other advanced disease were also excluded from the study.

Ethical clearance

Before beginning the study, we received ethical clearance from Velammal Medical College Hospital and Research Institute (approval number: VMCIEC/25/2021). Before enrollment in the trial, all participants provided written informed consent. Patient identity was kept private, and their responses were used solely for the study.

Data collection

During the initial visit, under local anesthesia, patients in the sclerotherapy group were placed in the left lateral position, and 3 to 5 mL of sodium tetradecyl sulfate was injected into a spot above the primary mass of hemorrhoid into the submucosa until elevation and pallor of the mucosa were observed. Similarly, in the rubber band ligation group, one rubber band was placed around each hemorrhoidal bundle on the rectal mucosa.

Patients were then followed up after three, six, and nine weeks. At each follow-up, the symptoms of bleeding, prolapse, pain, discharge, and pruritus/irritation were evaluated. Proctoscopy findings about the degree of hemorrhoids and any treatment-related problems were also recorded. Their treatment response was evaluated at follow-up visits and classified as complete when all hemorrhoids had disappeared or incomplete if any residual hemorrhoids were seen.

Patients were asked to assess the degree of symptomatic relief on a four-point Likert scale as excellent (patients who became completely asymptomatic), better (patients who had improvement of symptoms), same (patients who had persistence of symptoms without any improvement), and worse (patients whose symptoms worsened after treatment). Intraoperative pain during the treatment was scored on a Visual Analog Scale (VAS) ranging from 1 to 10, with 1 indicating no pain and 10 severe pain. If a patient remained symptomatic following the suggested treatment, the treatment was repeated up to three times. Patients who failed to respond three times were termed treatment failure, and surgery was recommended.

Data analysis

All data were entered into Microsoft Excel (Microsoft Corp., Redmond, WA, USA) and analyzed using SPSS version 20 (IBM Corp., Armonk, NY, USA) software. Descriptive characteristics were expressed as frequency and percentages. Using the Pearson chi-square test, the effectiveness between rubber band ligation and sclerotherapy was analyzed. P-values <0.05 were considered statistically significant.

## Results

A total of 200 patients were studied, of whom 100 belonged to the rubber band ligation treatment group and 100 to the sclerotherapy treatment group. The total number of male patients in the sclerotherapy group was 70 and the total number of female patients was 30. Almost 57 patients in the sclerotherapy group belonged to the 40-60-year age group, 34 to the 20-39-year age group, and nine were more than 60 years of age. The common comorbidities in the sclerotherapy group were diabetes (30%) and hypertension (18%), and 60 patients had no chronic comorbidities (Table 1). In the rubber band ligation group, the total number of male patients was 65 and the total number of female patients was 35. Almost 55 patients belonged to the 40-60-year age group, 29 to the 20-39-year age group, and 16 were more than 60 years of age. The common comorbidities in the rubber band ligation group were diabetes (38%) and hypertension (31%), and 49 patients had no chronic comorbidities (Table 1).

**Table 1:** General characteristics of the study participants (n = 200).

Characteristics	Sclerotherapy (N = 100)	Banding (N = 100)
Age group (years)		
20–39	34	29
40–60	57	55
>60	9	16
Gender		
Male	70	65
Female	30	35
Comorbidity		
Diabetes	30	38
Hypertension	18	31
Other comorbidities	11	8
None	60	49

Preoperative perianal pain was present among 34 patients in the banding group and 23 patients in the sclerotherapy group. Hemorrhoidal bleeding was present among 65 patients in the sclerotherapy group and 67 patients in the banding group. Nearly 55 patients experienced constipation in the banding group and 49 in the sclerotherapy group. The pile mass prolapse was present among nine patients in the banding group and six patients in the sclerotherapy group. Anal itching was present among five patients in the sclerotherapy group and 11 patients in the banding group. Almost 37 patients were anemic in the banding group and 28 were anemic in the sclerotherapy group. The intraoperative VAS score was severe for six patients in the sclerotherapy group and 11 in the banding group. It was mild for 59 patients in the sclerotherapy group and 45 in the banding group. Almost all preoperative symptoms were equally distributed among the sclerotherapy and banding groups, and none of the symptoms were significantly different between the two groups (Table 2).

**Table 2:** Distribution of preoperative symptoms and intraoperative pain in both treatment groups (n = 200). VAS = Visual Analog Scale

Preoperative symptoms	Sclerotherapy (N = 100)	Banding (N = 100)	P-value
Anal pain			
Yes	23	34	0.084
No	77	66	
Bleeding hemorrhoids			
Yes	65	67	0.765
No	35	33	
Constipation			
Yes	49	55	0.395
No	51	45	
Prolapsed hemorrhoids			
Yes	6	9	0.420
No	94	91	
Anal itching			
Yes	5	11	0.117
No	95	89	
Hemoglobin level			
Anemia	28	37	0.174
Normal	72	63	
Intraoperative VAS			
Mild	59	45	0.111
Moderate	35	44	
Severe	6	11	

Postoperative bleeding at three weeks was present among nine patients in the banding group and 18 patients in the sclerotherapy group, and it was statistically significant with a p-value <0.001. Hemorrhoidal prolapse at three weeks was present among 15 patients in the banding group and four patients in the sclerotherapy group, and it was statistically significant with a p-value of 0.007. Infection or discharge was present among 19 patients in the sclerotherapy group and eight patients in the banding group after three weeks of treatment, and it was statistically significant with a p-value of 0.022. Anal itching and pain were almost equally distributed in both treatment groups and were not statistically significant. At six and nine weeks after treatment, none of the above-mentioned symptoms were statistically significant (Table 3).

**Table 3:** Differences in the distribution of postoperative symptoms between both treatment groups (n = 200). SCL = sclerotherapy; RBL = rubber band ligation

Postoperative symptoms	At 3 weeks			At 6 weeks			At 9 weeks		
	SCL, N	RBL, N	P-value	SCL, N	RBL, N	P-value	SCL, N	RBL, N	P-value
Bleeding									
Positive	9	18	<0.001	7	11	0.322	4	4	1
Negative	91	82		93	89		96	96	
Prolapse									
Yes	4	15	0.007	4	11	0.060	4	10	0.096
No	96	85		96	89		96	90	
Pain									
Yes	54	59	0.475	39	45	0.390	33	35	0.765
No	46	41		61	55		67	65	
Infection/Discharge									
Yes	19	8	0.022	17	19	0.712	14	16	0.692
No	81	92		83	81		86	84	
Itch									
Yes	4	4	1	2	2	1	2	2	1
No	96	96		98	98		98	98	

About 94 patients showed complete treatment response in the sclerotherapy group after three weeks and only 77 patients showed complete treatment response in the banding group. This was statistically significant with a p-value <0.001. The treatment response was also significant after six weeks of treatment (p < 0.001) and nine weeks of treatment (p < 0.001). The patient response was similar in both treatment groups, with a majority reporting excellent treatment response, and there was a statistical difference between treatment groups (Table 4).

**Table 4:** Differences in the distribution of postoperative symptoms between both treatment groups (n = 200). SCL = sclerotherapy; RBL = rubber band ligation

Treatment response	At 3 weeks			At 6 weeks			At 9 weeks		
	SCL, N	RBL, N	P-value	SCL, N (%)	RBL, N (%)	P-value	SCL, N (%)	RBL, N (%)	P-value
Response to treatment									
Complete	94	77	<0.001	95	78	<0.001	96	81	<0.001
Incomplete	6	23		5	22		4	19	
Patient response									
Better	25	25	0.512	24	25	0.242	23	27	0.345
Excellent	60	52		63	55		67	60	
Same	9	13		8	7		7	5	
Worse	6	10		5	13		3	8	

## Discussion

The present study allocated 200 hemorrhoid patients into two treatment groups, namely, sclerotherapy and banding. They were compared in terms of demographic profile and primary and secondary outcomes. The primary outcome was treatment response assessed by the operating surgeon separately at three, six, and nine weeks after the procedure. The secondary outcomes were intraoperative VAS scores for pain and postoperative complications three, six, and nine weeks after the procedure.

The most common age group of our study participants was 40-60 years. This was similar to a study by Jehan et al. in which the most common age group was 45-60 years [10]. Another study by Bhuiya et al. also showed similar findings [4]. Hemorrhoids may be more common in middle-aged individuals than in old-aged individuals. Male patients were more common in our study group contributing to 67.5% of our study participants. The total male-to-female ratio in our study population was 2.07:1 which is similar to studies by Patel et al. (2.4:1) and Sohn et al. (2.75:1) [11,12].

Bleeding per rectum was the most common presenting symptom followed by constipation, perianal pain, and pile mass prolapse, while itching was the least common presenting symptom. A similar prevalence of symptoms was seen in studies by Jehan et al. (58%) and Murie et al. where bleeding per rectum was the most common presenting symptom [10,13].

All patients with grade two internal hemorrhoids had engorged anal cushions in 3, 7, and 11 positions variably. They were stratified after written informed consent into sclerotherapy and banding groups and underwent the procedures subsequently. Intraoperatively, pain during the procedure was assessed through VAS scores which ranged from 1 to 10. Scores ranging from 1 to 3 were classified as mild, 4 to 6 as moderate, and >6 as severe. In our study groups, intraoperative severe pain was more commonly associated with banding (11%) than with sclerotherapy (6%). This result was similar to studies done by Shah et al. and Tchirkow et al. However, the result was in contrast to the study by Awad et al. [14-16].

In the present study, rubber band ligation was significantly associated with the complication of recurrent mass prolapse. This result is similar to the study by Santos et al. but in contrast to studies by Shah et al. and Laghari et al. [17,18]. Similarly, all other complications were also comparatively high in the banding group when compared to the sclerotherapy treatment group. The reason for this difference may be due to multiple factors such as the individual patient’s health status, the size and location of the hemorrhoids, and the skill and experience of the healthcare provider performing the procedure.

Although the present study had an adequate sample size, the study has certain limitations. The study was conducted in a single tertiary care hospital and the sample was not representative of the broader population. The choice of treatment was decided by the participant, and without random assignment to treatment and control groups, there is a higher risk of selection bias. Certain outcomes were based on patient self-reports (e.g., pain) and were less reliable than objective measures. Moreover, factors such as variations in surgical technique, surgeon experience, postoperative care, and patient adherence to postoperative protocols can influence outcomes.

## Conclusions

Intraoperative and immediate postoperative pain was higher in the rubber band ligation group than in the sclerotherapy group. Post-procedure complications were more commonly seen in the rubber band ligation group than in the sclerotherapy group at various weeks of the procedure. Overall, the sclerotherapy group was associated with fewer postoperative complications, more excellent patient response, and a more complete response to treatment than the rubber band ligation group. However, the sclerotherapy procedure was also associated with postoperative complications such as infection, anal bleeding, and itching more commonly or equally as the rubber band ligation group. With these inferences, we can consider the sclerotherapy procedure to be superior to rubber band ligation in terms of fewer complications and more efficacy in our study. However, more robust interventional studies and meta-analyses are required to sufficiently justify the hypothesis.
